# The International Dental Federation

**Published:** 1903-02-15

**Authors:** H. A. Smith


### THE INTERNATIONAL DENTAL FEDERATION.

BY H. A. SMITH, D.D.S.

Substance of Remarks made at the recent meeting of the Ohio State Dental Society.

The International Federation and Commission of Education was organized at the Dental Congress in Paris in 1900, but its first meeting was held in England last year, and the second meeting has just been held in Stockholm, Sweden, during the past summer. The next meeting will be held in Madrid in connection with the International Congress of Medicine which convenes in that city in April, 1903. The fourth session will probably be held in St. Louis, in connection with a World's Dental Congress, at the time of the World's Fair in 1904.

The object of this organization is educational in a sense. So far it seems to be the desire of its promoters that a comity of educational methods and standards may be made international. Technical sciences have as yet not received much consideration, as most of the time has been taken up with considering educational methods and standards. But it is probable that at a near time steps will be taken to at least gather historical statistics as to the progress of every phase of scientific progress throughout the world. At the recent meeting there were present representatives from almost every civilized nation. Some countries were liberally represented. This country was well represented in numbers and talent as well. England was not so well represented as was expected in view of the meeting there last year.

The question of a universal method of training dentists is the vital one. We hardly realize the difference in the systems of dental education in this country and abroad. American methods have made a deep impression on the men of other countries. Abroad a dental student is trained in a medical college. He must take two or three years in medicine, and much that pertains to dentistry he learns from a preceptor outside. The body which educates him pays no attention to dentistry. He goes before a medical board to be examined for a license to practice dentistry. He has no degree; they give him none. It is because of such methods that American dentists excel. The purpose of this federation is to bring about a uniform system of dental education. The prospects seem to me not very encouraging. All that has been done was to appoint a committee on curriculum of dental studies. There is also a committee on oral hygiene which is interested in the instruction of the masses.

At the meeting the subject of qualifications for admission occupied more time than anything else. What shall be the qualifications of the applicant for admission to the institutions throughout the world? Naturally the people of Europe felt that their standard was good. Germany wished to have a standard equivalent to that for admission to schools of law and medicine. Finally a resolution was adopted to the effect that students seeking admission to all dental schools should have an education equivalent to admission to the schools of law and medicine in countires where the schools are regulated by the government. In countries where educational matters were not under government control, the standard should be the same sa where these matters were regulated by the government. This makes it impossible for any school having federation membership to make the standard lower than any other schools in the federation.

The Ecole Odontaire in Paris has already taken up the four year course, and adopted the best American and European methods.

There was some dissension in the Executive Council as to the proposition to hold the fourth International Dental Congress at St. Louis in 1904. The German and English members thought it was too soon after the third congress to make it possible to organize a Congress of any international interest or value. The majority of the committee however favored the plan. It will probably be finally considered at Madrid in April.

